# Efficient BiVO_4_ Photoanodes by Postsynthetic Treatment: Remarkable Improvements in Photoelectrochemical Performance from Facile Borate Modification

**DOI:** 10.1002/anie.201911303

**Published:** 2019-11-08

**Authors:** Qijun Meng, Biaobiao Zhang, Lizhou Fan, Haidong Liu, Mario Valvo, Kristina Edström, Maria Cuartero, Roland de Marco, Gaston A. Crespo, Licheng Sun

**Affiliations:** ^1^ Department of Chemistry School of Engineering Sciences in Chemistry Biotechnology and Health KTH Royal Institute of Technology 10044 Stockholm Sweden; ^2^ Department of Chemistry Ångström Laboratory Uppsala University 75120 Uppsala Sweden; ^3^ Faculty of Science, Health, Education and Engineering University of the Sunshine Coast 90 Sippy Dows Drive Sippy Downs Queensland 4556 Australia; ^4^ School of Chemistry and Molecular Biosciences The University of Queensland Brisbane Queensland 4072 Australia; ^5^ State Key Laboratory of Fine Chemicals Institute of Artificial Photosynthesis DUT-KTH Joint Education and Research Center on Molecular Devices Dalian University of Technology 116024 Dalian China

**Keywords:** artificial photosynthesis, BiVO_4_, borate, photoelectrochemical cells, water oxidation

## Abstract

Water‐splitting photoanodes based on semiconductor materials typically require a dopant in the structure and co‐catalysts on the surface to overcome the problems of charge recombination and high catalytic barrier. Unlike these conventional strategies, a simple treatment is reported that involves soaking a sample of pristine BiVO_4_ in a borate buffer solution. This modifies the catalytic local environment of BiVO_4_ by the introduction of a borate moiety at the molecular level. The self‐anchored borate plays the role of a passivator in reducing the surface charge recombination as well as that of a ligand in modifying the catalytic site to facilitate faster water oxidation. The modified BiVO_4_ photoanode, without typical doping or catalyst modification, achieved a photocurrent density of 3.5 mA cm^−2^ at 1.23 V and a cathodically shifted onset potential of 250 mV. This work provides an extremely simple method to improve the intrinsic photoelectrochemical performance of BiVO_4_ photoanodes.

## Graphical abstract

**A BiVO_4_ photoanode** was modified with borate at the molecular level (B‐BiVO_4_) by a simple immersion method, giving rise to increased photocurrent and decreased onset potential for water oxidation, which is comparable to loading a water‐oxidation co‐catalyst. The self‐anchored borate plays the role of passivator in decreasing the surface charge recombination and also a ligand in modifying the catalytic site to facilitate faster water oxidation.

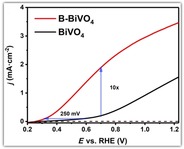

## Introduction

Water splitting by photoelectrochemical (PEC) cells is one of the most promising ways to obtain a renewable H_2_ fuel.^1^ Since electrochemical photolysis of water at a TiO_2_ photoanode was reported by Fujishima and Honda in 1972,^2^ metal oxide based semiconductors have become attractive materials for photocatalysis and PEC cells.^3^ An ideal semiconductor applicable for a PEC cell requires a suitable band gap to utilize a significant portion of the solar spectrum, an effective charge separation in the bulk, an efficient charge transfer at the semiconductor/electrolyte interface, and a long‐term stability in aqueous media.^4^ Among the metal oxide based semiconductors, monoclinic bismuth vanadate (BiVO_4_) is considered the most promising owing to its suitable band gap (ca. 2.4 eV) that enables it to absorb about 11 % of the visible light spectrum, its long carrier lifetime (ca. 40 ns), low cost, and good stability.^5^ Under the standard AM 1.5 G sunlight illumination, the theoretical photocurrent density of BiVO_4_ is estimated to reach a maximum of 7.5 mA cm^−2^, resulting in a solar‐to‐hydrogen conversion efficiency of close to 9.2 %.4b, 6

However, the PEC performance of pure BiVO_4_ photoanode is greatly limited by its low carrier mobility (ca. 4×10^−2^ cm^2^ V^−1^ s^−1^), short hole‐diffusion length (ca. 100 nm), and slow water oxidation kinetics.^7^ Plenty of approaches have been attempted to overcome these limitations, including element doping,^8^ morphology engineering,^9^ heterostructure formation,^10^ oxygen evolution catalysts (OECs)‐layer loading,^11^ crystal facet engineering,^12^ plasmonic enhancement,^13^ and combinations thereof. However, the efficiency of BiVO_4_ photoanodes is still far from an application level.5a Beside these well‐studied techniques, a series of postsynthetic treatments, a concept proposed by Smith and Stefik, have recently emerged as a simple and effective strategy to enhance the intrinsic photocatalytic activity of BiVO_4_ photoanodes.^14^ Instead of requiring the use of additional materials, such posttreatments stand out as methods to change the defect chemistry, both at the surface and in the bulk of BiVO_4_. It provides new mechanisms and opportunities to understand and enhance the intrinsic properties of BiVO_4_ photoanodes for higher PEC performance.

To date, a variety of postsynthetic modifications have been reported, including annealing under H_2_ or N_2_,^15^ illumination (that is, photocharging),^16^ UV curing,^17^ electrochemical treatment,^18^ acid vapor etching,12b Li/EDA (ethylenediamine) solution treatment,^19^ and so on. Herein, we found an extremely facile postsynthetic treatment for the improvement of BiVO_4_ photoanodes: modifying the BiVO_4_ electrodes with a borate species at the molecular level. The treated BiVO_4_ photoanodes (denoted as B‐BiVO_4_) consistently exhibit excellent PEC performance for water oxidation under AM 1.5 G illumination, with a near tenfold enhancement of photocurrent at 0.7 V_RHE_ and a cathodic shift of the onset potential by 250 mV. A series of control experiments were performed; detailed physical characterizations, electrochemical impedance spectroscopy (EIS), and kinetic isotope effect (KIE) studies were conducted to reveal the significant role played by the addition of the borate moiety.

## Results and Discussion

**Figure 1:**
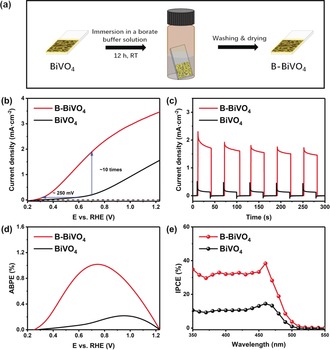
a) B‐BiVO_4_ photoanode preparation. b) Photocurrent–potential (*J*–*V*) curves of bare BiVO_4_ and B‐BiVO_4_ photoanodes under AM 1.5 G simulated sunlight at 100 mW cm^−2^ in a 0.5 m borate buffer (pH 9.3). Scan rate: 10 mV s^−1^. c) Transient photocurrents for BiVO_4_ and B‐BiVO_4_ photoanodes measured at 0.7 V_RHE_. d) Applied bias photon‐to‐current efficiencies (ABPEs) of BiVO_4_ and B‐BiVO_4_ photoanodes. e) Incident photon‐to‐current efficiencies (IPCEs) of BiVO_4_ and B‐BiVO_4_ photoanodes at 0.7 V_RHE_.

Nanoporous BiVO_4_ photoanodes were prepared according to an established method, with a few minor modifications.5c A typical worm‐like nanostructure of the resulting BiVO_4_ with a thickness of about 600 nm is shown in the SEM images (Supporting Information, Figure S1a,b). The monoclinic phase and a band gap of 2.42 eV are indicated by X‐ray diffraction and UV/Vis absorption spectra, respectively (Supporting Information, Figure S1c,d). Borate modification of the BiVO_4_ photoanode was performed by simply dipping the pristine BiVO_4_ in a 0.5 m borate buffer solution (pH 9.3) in a capped dark brown bottle (Figure 1 a). After 12 h, the treated BiVO_4_ electrode was removed from the borate solution and rinsed with Milli‐Q water to afford B‐BiVO_4_.

PEC performances of pristine BiVO_4_ and B‐BiVO_4_ were monitored in a three‐electrode cell, with 0.5 m borate buffer (pH 9.3) as electrolyte, under simulated sunlight illumination (AM 1.5 G, 100 mW cm^−2^). Pristine BiVO_4_ showed an onset potential of 0.57 V (defined at 0.1 mA cm^−2^ photocurrent density) and a maximum photocurrent density of only 1.6 mA cm^−2^ at 1.23 V vs. a reversible hydrogen electrode (RHE; Figure 1 b). Surprisingly, a highly improved photocurrent density was exhibited by B‐BiVO_4_, reaching 3.5 mA cm^−2^ at 1.23 V. The onset potential cathodically shifted to 0.32 V. Photocurrent density of B‐BiVO_4_ at 0.7 V is approximately ten times higher than that of the pristine BiVO_4_. The significantly enhanced PEC performance of B‐BiVO_4_ was further confirmed by the transient photocurrent (Figure 1 c), applied bias photon‐to‐current efficiency (ABPE, Figure 1 d), and incident photon‐to‐current conversion efficiency (IPCE) measurements (Figure 1 e). A maximum ABPE of 1.1 % was obtained by B‐BiVO_4_. IPCE of B‐BiVO_4_ at 0.7 V showed a universal double increment compared to the pristine BiVO_4_ and reached a maximum of 38 % at a wavelength of 460 nm.

The B‐BiVO_4_ photoanode, without the typical dopant or any co‐catalyst, displayed superior PEC performance even when compared to many doped and catalyst‐modified BiVO_4_ photoanodes (Supporting Information, Table S1). In general, state‐of‐the‐art performance of pristine BiVO_4_ is about 1.5 mA cm^−2^ at 1.23 V.5c For most of postsynthetically treated BiVO_4_ photoanodes, the photocurrent densities are only around 2.5 mA cm^−2^ (for example, 2.8, 2.4, and 2.5 mA cm^−2^ for N_2_,15a H_2_,15b and electrochemical treatments^18^ of BiVO_4_, respectively). Only two kinds of undoped and uncatalyzed BiVO_4_ photoanodes, reported recently, exhibited performances comparable to B‐BiVO_4_ (Supporting Information, Table S2). The photocharged BiVO_4_ photoanodes, investigated by Smith and co‐workers, achieved a photocurrent density of 4.3 mA cm^−2^ at 1.23 V.16a Cho and Zheng developed [001]‐oriented BiVO_4_ photoanodes with photocurrent density of 3.9 mA cm^−2^ at 1.23 V.12b The B‐BiVO_4_ displayed top level PEC performance among the undoped and uncatalyzed BiVO_4_ photoanodes. Additionally, the treatment method used in this case is more facile than other postsynthetic treatment methods.

Regarding the stability of B‐BiVO_4_ under an open‐circuit condition, when B‐BiVO_4_ was stored under air for 24 h, the PEC performance showed only a small decrease (Supporting Information, Figure S2); when B‐BiVO_4_ was stored in Milli‐Q water overnight, the PEC performance kept approximately 85 % of its incipient performance (Supporting Information, Figure S3). These observations distinguish B‐BiVO_4_ from the BiVO_4_ after photocharging treatment, where the photocharged BiVO_4_ totally lost its increment of PEC performance when stored in dark overnight in buffer solution,16b indicating that a different underlying mechanism is responsible for the improvement in the PEC performance of B‐BiVO_4_.

**Figure 2:**
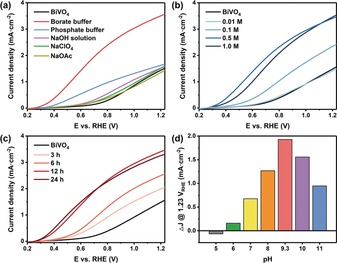
*J*–*V* curves for BiVO_4_ and B‐BiVO_4_ photoanodes treated a) with different salt solutions at pH 9.3; b) in different concentrations of borate buffer at pH 9.3; c) in a 0.5 m borate buffer at pH 9.3 for different durations. d) Increments of photocurrents at 1.23 V_RHE_ of B‐BiVO_4_ photoanodes treated with a 0.5 m borate buffer at different pH values compared to the bare BiVO_4_.

To investigate this underlying mechanism, we firstly established, by means of a series of control experiments on the immersion treatment, that the remarkable effect is indeed caused by the involvement of the borate species. The possibility that the improvement in PEC performance is due to the basic pH condition can be safely excluded as no obvious change in the photocurrent density is observed when the pristine BiVO_4_ is soaked in a NaOH aqueous solution (pH 9.3) instead of the borate solution (Figure 2 a). Regarding the effect of salt ions, a treatment with neither NaOAc nor NaClO_4_ solution brings in an improvement in the photocurrent of BiVO_4_. The bare BiVO_4_, treated with a phosphate buffer, showed some visible enhancement of PEC performance, but it was still far less than B‐BiVO_4_.

Furthermore, the borate treatment itself was studied in greater detail by changing the borate concentration, immersion time, temperature, and pH value of the borate solution. PEC performances of the corresponding B‐BiVO_4_ photoanode markedly rose with the increase in the borate concentration under the same soaking duration (Figure 2 b). PEC performances of the resulting B‐BiVO_4_ treated in the same borate solution improved with respect to the immersion time (Figure 2 c) during the first 12 h. Extension of the immersion time over 12 h led to negligible improvement, indicating that the full transformation of the pristine BiVO_4_ to B‐BiVO_4_ was completed in the stipulated time. Interestingly, it was found that the borate treatment can be considerably accelerated by increasing the reaction temperature (Supporting Information, Figure S4); B‐BiVO_4_ with the best PEC performance can be generated after only 25 min of treatment at 100 °C. Especially noteworthy is the fact that the effect of the modification is highly dependent on the pH of the borate solution. The highest improvement was achieved by the treatment with a borate solution in the pH range of 9 to 10, approaching boric acid p*K*_a_ of 9.24 (Figure 2 d; Supporting Information, Figure S5). The correlation between the enhancing effect and the pH of the borate solution suggests that [B(OH)_4_]^−^, the conjugate base of H_3_BO_3_, may be directly involved in modifying the BiVO_4_ sample and also plays a pivotal role in the PEC performance improvement. These results therefore confirm that the improvement of PEC performance resulted from the modification by the borate species, most likely to be [B(OH)_4_]^−^ with a tetrahedral geometry.

**Figure 3:**
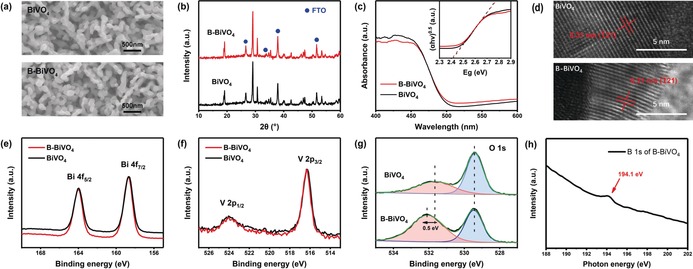
a) SEM images, b) X‐ray diffraction (XRD) spectra, and c) UV/Vis diffuse spectra of BiVO_4_ and B‐BiVO_4_ photoanodes. Inset: Tauc plots of BiVO_4_ and B‐BiVO_4_. d) HRTEM images of the bare BiVO_4_ and B‐BiVO_4_ photoanodes, respectively. e) Bi 4f, f) V 2p, and g) O 1s XPS spectra for bare BiVO_4_ and B‐BiVO_4_ photoanodes, respectively. h) B 1 s NEXAFS edge spectrum for the B‐treated BiVO_4_ sample.

To explore the structural changes of the BiVO_4_ film after the borate modification, physical characterizations were conducted for both the pristine and modified BiVO_4_. However, SEM images (Figure 3 a) and XRD patterns (Figure 3 b) of B‐BiVO_4_ show no noticeable differences compared to that of the bare BiVO_4_. The UV/Vis absorption spectra of both the modified and pristine BiVO_4_ also exhibit similar absorbance edges at approximately 520 nm, indicating the similar band gap of about 2.4 eV (Figure 3 c). These demonstrate that the nature of the bulk of B‐BiVO_4_, for example, structure and absorbance, remain unchanged.

Therefore, we can conclude that the alteration of the BiVO_4_ film, induced by the borate modification, happens owing to the changes on the surface. Raman spectroscopy, high‐resolution transmission electron microscopy (HRTEM), X‐ray photoelectron spectroscopy (XPS), and near‐edge X‐ray absorption fine structure (NEXAFS) spectroscopy, all of which are powerful techniques for surface characterization, were employed to explore structural details of the surface changes by the borate modification. Unfortunately, the Raman spectra of the pristine BiVO_4_ and B‐BiVO_4_ were found to be superimposable, showing no identifiable structural changes (Supporting Information, Figure S6). No obvious interface or newly generated nanolayer was observed from the HRTEM images either (Figure 3 d). The XPS spectra of both species exhibited typical O 1s, V 2p, and Bi 4f peaks (Supporting Information, Figure S7). The Bi 4f and V 2p peaks, and the O 1s peak at 529.4 eV, displayed negligible shifts before and after the borate treatment (Figures 3 e–g). The only obvious change is that the O 1s peak at 531.6 eV, which is commonly attributed to chemisorbed −OH groups, shifts to 532.1 eV, with an evidently higher density of such groups (Figure 3 g). This change can be a sign of an increase in chemisorbed ‐OH groups due to the absorption of [B(OH)_4_]^−^ or −OH or both.^20^ However, it should be noted that surface contamination (−CO and −CO_2_) may also cause changes in the O 1s peak.^21^

It has been clearly demonstrated earlier that a borate moiety is involved in the modification of the surface of the BiVO_4_ film to afford an efficient B‐BiVO_4_ species. Unfortunately, most of the above characterization methods failed to show the nature of the exact changes. Even a boron signal could not be identified in the elemental analysis by XPS (Supporting Information, Figure S7) or HRTEM‐EDS (EDS corresponds to energy‐dispersive X‐ray spectroscopy; Supporting Information, Figure S8). However, this is not a factual contradiction, because boron is very light element. As it is in a system with heavy metal, the detection limits of both these techniques are very high.^22^ It is difficult to detect a B signal when its content is not abundant in the sample. Even for a typical boron‐doped BiVO_4_ with B‐compositions of 3 % and 10 %,^20^, ^23^ the observed B signals are very weak, indicating the level of a detection limit. In comparison, the amount of surface absorbed borate in this case can be orders of magnitude lower. This should explain the failure in detecting a B signal. The missing B signal in the regular physical characterization, in effect, indicates that the borate modification of the BiVO_4_ surface is at a molecular level with an extremely low borate concentration.

To display the presence of trace amount of borate on BiVO_4_ surface, we conducted a more sensitive characterization, the NEXAFS measurements by using low‐energy secondary electrons. The NEXAFS B 1s edge spectrum displayed that there may have been a trace of B at the surface of the B‐treated BiVO_4_ sample, as revealed by a small peak at approximately 194.1 eV owing to the boric acid/borate species in Figure 3 h.^24^ NEXAFS measurements were accomplished using low energy secondary electrons of about 14 eV (more precisely over a 13–15 eV range), noting that the inelastic mean free path (IMFP) of the detected secondary electrons, which is related to the escape depth and sampling depth of NEXAFS, is 3.6 nm at this electron energy with inorganic materials.^25^ Accordingly, the NEXAFS data pertain to the sample surface indicating the presence of B at a trace level. It is unsurprising that NEXAFS located a trace of B in the treated sample, although XPS were unable to detect B signal. Indeed, this is not a precedent in the NEXAFS detection of trace elements owing to the enhanced sensitivity of NEXAFS. For example, NEXAFS of the Fe L‐edge yielded high‐quality spectra with the detection of Fe^II^/Fe^III^ states at an Fe depleted iron chalcogenide surface since the photoabsorption cross‐section increased by several orders of magnitude, substantially boosting the analytical sensitivity of NEXAFS when the incident beam energy approached and resonated with the Fe L‐edge.^26^

**Figure 4:**
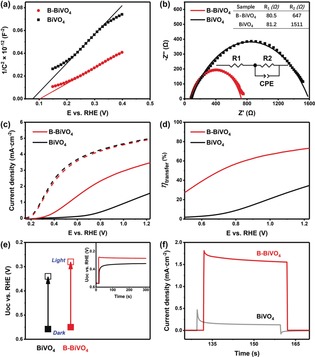
a) Mott–Schottky plots of BiVO_4_ and B‐BiVO_4_ photoanodes measured in a 0.5 m borate buffer at pH 9.3 in dark. b) Electrochemical impedance spectra (EIS) of BiVO_4_ and B‐BiVO_4_ photoanodes measured at 0.7 V_RHE_. c) *J*–*V* curves of BiVO_4_ and B‐BiVO_4_ photoanodes for sulfite oxidation measured in a 0.5 m borate buffer (pH 9.3) containing 0.5 m Na_2_SO_3_ (hole scavenger). d) Charge transfer efficiencies at the semiconductor/electrolyte interface (*η*_transfer_) of BiVO_4_ and B‐BiVO_4_ photoanodes. e) Open circuit potentials (U_OC_) of BiVO_4_ and B‐BiVO_4_ photoanodes under dark (solid) and illumination (hollow); inset: transient photovoltage response within immediate illumination. f) Transient photocurrents measured at 0.7 V_RHE_ for BiVO_4_ and B‐BiVO_4_.

To further reveal the underlying mechanism of the dramatic effect induced by the borate modification, we thoroughly investigated the photogenerated carrier transfer kinetics of BiVO_4_ before and after the borate treatment. Mott–Schottky curves of both samples show positive slopes, as expected, for the n‐type semiconductors (Figure 4 a). Based on the slope of the Mott–Schottky curves, carrier density increment of B‐BiVO_4_, compared to that of the pristine BiVO_4_, is negligible. The flat band potential (intercept on *x* axis) of the bare BiVO_4_ anodically shifts by only a small value of 25 mV. Moreover, an anodic shift cannot contribute to the negative shift of the photocurrent onset potential of B‐BiVO_4_ for water oxidation. The Mott–Schottky analysis again demonstrates that the bulk properties of BiVO_4_ are not affected by the borate modification. Electrochemical impedance spectroscopy (EIS) measurements show that the B‐BiVO_4_ photoanodes have the same series resistance *R*_s_ but a much smaller interfacial charge transfer resistance *R*_ct_ as that of the pristine BiVO_4_ (Figure 4 b), indicating that the improvement in photocurrent density of B‐BiVO_4_ can be attributed to the enhanced surface charge transfer rather than to the bulk charge transport.

More precisely, the contributions of the increased photocurrent density (*J*), which is determined by three fundamental components [given by Eq. (1)], namely light absorption (represented as *J*_abs_), charge transport efficiency in the bulk (*η*_transport_), and charge transfer at the semiconductor/electrolyte interface for water oxidation (*η*_transfer_), were studied to confirm the identification of the key factors for the high PEC performance observed in the case of B‐BiVO_4_.

(1) $$J=J{}_{\mathrm{abs}}\;\eta {}_{\mathrm{transport}}\;\eta {}_{\mathrm{transfer}}$$

The borate treatment has trivial effect on *J*_abs_, because the unmodified BiVO_4_ and B‐BiVO_4_ have comparable light absorption properties, as shown by the similar UV/Vis absorption spectra for both. The *η*_transport_ and *η*_transfer_ were separately evaluated by employing a conventional hole‐scavenger method. Figure 4 c shows the *J*–*V* curves for the pristine BiVO_4_ and B‐BiVO_4_ photoanodes, determined in the electrolyte with and without a hole‐scavenger, Na_2_SO_3_. In contrary to the differences in PEC performances for water oxidation, the pristine BiVO_4_ and B‐BiVO_4_ exhibited comparable photocurrent density when sufficient Na_2_SO_3_ was introduced in the electrolyte. Considering that *J*_abs_ is the same for both samples, it is rational to deduce that B‐BiVO_4_ has the same *η*_transport_ as the pristine BiVO_4_. In contrast, *η*_transfer_ of B‐BiVO_4_, as shown in Figure 4 d, is at least two‐fold higher than that of the pristine BiVO_4_, depending on the applied potential.

Finally, we found out that the immensely increased *η*_transfer_ (that is, surface catalytic efficiency) is the key factor in the observed improvement in PEC performances of B‐BiVO_4_ after the borate treatment. Three factors can be responsible for an increase in *η*_transfer_, including a larger surface area, suppressed surface charge trapping, and an accelerated catalytic rate of water oxidation reaction. First, the surface areas of the BiVO_4_ photoanode before and after the borate treatment were evaluated by electrochemical capacitance measurements (Supporting Information, Figure S9). Electrochemically active surface areas (EASA) of the pristine BiVO_4_ and B‐BiVO_4_ were found to be similar, which rules out its contribution to the higher *η*_transfer_. The case of surface charge trapping was investigated by measuring the open‐circuit voltage (*U*_oc_).11d When BiVO_4_ is immersed in the electrolyte, the illumination induced increment in *U*_oc_ depends on the photogenerated carrier density, which results in a new quasi‐Fermi level. The photovoltage for B‐BiVO_4_ was detected as 0.27 V, which was 50 mV higher than that detected for the bare BiVO_4_, indicating the suppression of surface charge trapping on B‐BiVO_4_ (Figure 4 e). The 50 mV of photovoltage difference between BiVO_4_ and B‐BiVO_4_ is much less than the 250 mV cathodic shift in the onset potential for water oxidation. Therefore, suppression of surface charge trapping is one of the factors that must have played a role in the enhancement of *η*_transfer_ of B‐BiVO_4_.

Since the *J*–*V* curve for the B‐BiVO_4_ photoanode, determined with a hole‐scavenger, did not show any cathodic shift, while its *J*–*V* curve for water oxidation cathodically shifted 250 mV (Figure 4 c), it is obvious that the rate of water oxidation on the B‐BiVO_4_ photoanode was enhanced tremendously, which is the other important factor facilitating surface charge transfer in the case of B‐BiVO_4_. The faster water oxidation on the B‐BiVO_4_ surface can be further established by the study of photocurrent transients. Light on–off cycles in chopped light chronoamperometry is usually accompanied by photocurrent transient spikes, caused by the discrepancy between the fast carrier generation and slow surface reaction dynamics.8b, 11d, 27 The spikes for the B‐BiVO_4_ photoanodes are much smaller compared to that of the bare BiVO_4_ ones; moreover, no charge accumulation was found for B‐BiVO_4_, as shown by the damped‐current during light‐off (Figure 4 f). These observations demonstrate that the borate modification accelerated the catalytic rate of water oxidation on the modified BiVO_4_ surface. Indeed, the dramatic effect of surface modification on photocatalytic performance have been studied for other bismuth‐based semicondutors.^28^

**Figure 5:**
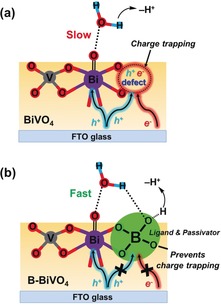
Illustration of the proposed mechanism for water oxidation on the surface of a) pristine BiVO_4_ and b) B‐BiVO_4_.

Based on the control experiments, physical characterizations, and carrier transfer kinetics studies, we propose that the immersion treatment in borate buffer solution is indeed a spontaneous process in which the tetrahedral [B(OH)_4_]^−^ gradually interacts with the active site (that is, defect) on the BiVO_4_ surface (Figure 5). The most likely sites for the tetrahedral [B(OH)_4_]^−^ are the defects formed as a result of vanadium loss.^23^, ^29^ The adsorbed [B(OH)_4_]^−^ may act as a passivator to reduce charge recombination22a and to facilitate extraction of holes to the surface.^30^ More importantly, the anchoring of the borate moiety at the catalytic active site significantly accelerated the catalytic rate of water oxidation. The role played by the self‐anchored borate can be considered as a ligand effect at the catalytic site on the BiVO_4_ surface. It can modify the electronic configuration of the bismuth catalytic site and consequently, accelerate the O−O bond formation rate. At the same time, the anchored borate, as an internal base, can also assist the concerted proton‐electron transfer, which has been shown to be essential for water oxidation by molecular catalysts,^31^ metal oxides,^32^ and semiconductor photoanodes.^33^ KIE studies of the pristine BiVO_4_ and B‐BiVO_4_ photoanodes indicated that proton transfer is involved in the rate determining step (RDS) because a KIE value of approximately 2.6 was observed for the pristine BiVO_4_ with low bias; the anchored borate evidently facilitated proton transfer in the RDS, with a much smaller KIE value of around 1.5 determined for the B‐BiVO_4_ photoanode (Supporting Information, Figure S10).

The stability of B‐BiVO_4_ under PEC test was evaluated by multiple cycles of linear sweep voltammetry (LSV) under illumination (Supporting Information, Figure S11) and photocurrent–time measurements (Supporting Information, Figure S12). PEC performance of B‐BiVO_4_ gradually decreased during 20 cycles of LSV. After 20 min of photoelectrolysis with 1.0 V bias, B‐BiVO_4_ lost approximately 35 % of the initial photocurrent. In a separate experiment, a Faradaic efficiency of 91 % for oxygen evolution by the B‐BiVO_4_ was calculated based on the record of the moles of electrons passing through and the determination of the amounts of evolved oxygen (Supporting Information, Figure S13). The deactivation of B‐BiVO_4_ can be induced by photocorrosion11b, 34 or desorption of the borate from the photocharged surface of B‐BiVO_4_ or both. Deactivation of the bare BiVO_4_, without a catalytic or passivating layer, has been widely observed under long‐term PEC tests.11b, 35 Interestingly, when the process of borate treatment was repeated on B‐BiVO_4_ after 20 cycles of LSV scanning, similar PEC performances as that from a freshly‐prepared B‐BiVO_4_ can be obtained again (Supporting Information, Figure S14). This self‐recovery process can be repeated several times and projects borate treatment as a possible strategy to produce self‐healing PEC cells, which can work during daytime and recover during the night (Supporting Information, Figure S15). Furthermore, modifying the B‐BiVO_4_ with co‐catalyst can further increase its photocurrent density for water oxidation and dramatically improve the stability. These related studies are ongoing in our group.

## Conclusion

In summary, we reported the remarkable effect of modifying a BiVO_4_ surface with borate by a simple immersion method, leading to a significant increase in photocurrent as well as a decrease in the onset potential for water oxidation, which is comparable to the effect of loading a water‐oxidation co‐catalyst. Detailed characterizations and carrier transfer kinetics investigations indicated that the adsorption of tetrahedral [B(OH)_4_]^−^ species near the active sites results in a molecular level modification. This acts as a regulating ligand and passivator, playing an important role in accelerating water‐oxidation rate and reducing charge trapping on the BiVO_4_ surface. The post‐synthetic borate treatment proposed in this work provides new opportunities to understand and improve the PEC performance of BiVO_4_ photoanodes. The method of small molecule modification can also be widely developed for improving the property of material‐based catalysts and photocatalysts.

## Conflict of interest

The authors declare no conflict of interest.

## Supporting information

As a service to our authors and readers, this journal provides supporting information supplied by the authors. Such materials are peer reviewed and may be re‐organized for online delivery, but are not copy‐edited or typeset. Technical support issues arising from supporting information (other than missing files) should be addressed to the authors.

Supplementary
