# Supplementary material for: Efficient BiVO4 Photoanodes by Postsynthetic Treatment: Remarkable Improvements in Photoelectrochemical Performance from Facile Borate Modification
Source: Angew Chem Int Ed Engl. 2019 Nov 8;58(52):19027–33. doi: 10.1002/anie.201911303 (PMC6973097; doi:10.1002/anie.201911303)
Supplement: Supplementary file 1 — Supplementary [file ANIE-58-19027-s001.pdf]

Angewandte Chemie
Eine Zeitschrift der Gesellschaft Deutscher Chemiker

Supporting Information

Efficient $BiVO_4$ Photoanodes by Postsynthetic Treatment: Remarkable Improvements in Photoelectrochemical Performance from Facile Borate Modification

Qijun Meng, Biaobiao Zhang, Lizhou Fan, Haidong Liu, Mario Valvo, Kristina Edström, Maria Cuartero, Roland de Marco, Gaston A. Crespo, and Licheng Sun*

anie_201911303_sm_miscellaneous_information.pdf

Table of Contents

Experimental Procedures 2
Materials 2
Preparation of $BiVO_4$ photoanodes 2
Photoelectrochemical Measurements 2
Physical Characterization 2
Figure S1. 3
Figure S2. 4
Figure S3. 5
Figure S4. 6
Figure S5. 7
Figure S6. 8
Figure S7. 9
Figure S8. 10
Figure S9. 11
Figure S10. .... **Error! Bookmark not defined.**12
Figure S11. 12
Figure S12. 13
Figure S13. 14
Figure S14. 16
Figure S15. 17
Table S1. 19
Table S2. 19
References 20
Author Contributions 21

Experimental Procedures

Materials

Bismuth nitrate pentahydrate ($Bi(NO_3)_3 \cdot 5H_2O$, 98%), p-benzoquinone (98%), potassium iodide (KI, 99%), vanadyl acetylacetonate ($VO(acac)_2$, 98%), sodium sulfite (Na_2SO_3, 98%), Nitric acid solution(HNO_3, 70%), ethanol absolute (EtOH), dimethyl sulfoxide (DMSO), deuterium oxide (D_2O), deuterium sodium oxide (NaOD, 40 wt. %), and deuterium chloride (DCl, 35 wt. %) solution were purchased from Sigma-Aldrich and used as received without further purification. Ultra-pure water (18.2 $M\Omega \cdot cm^{-1}$) supplied by a Milli-Q system (Merck Millipore) was used in all experiments. Fluorine-doped tin oxide (FTO) coated glass substrates were purchased from Pilkington (~8 $\Omega \cdot cm^{-2}$) and were successively cleaned in Milli-Q water, ethanol and acetone by ultrasonic bath.

Preparation of $BiVO_4$ photoanodes

The nanoporous $BiVO_4$ photoanodes were prepared according to an established method with some modifications.[1] In brief, a 0.04 M $Bi(NO_3)_3$ was dissolved into 50 mL of pH 1.7 HNO_3 aqueous solution with 5 min sonification. Then 0.4 M KI was added to this solution with further 5 min sonification. Afterwards, a 20 mL of ethanol containing 0.23 M p-benzoquinone was mixed to the above solution with vigorously stirring. The electrodeposition of BiOI precursor was carried out potentiostatically at -0.1V vs. Ag/AgCl for 3 min at room temperature, using a typical three-electrode cell: an FTO working electrode (WE), a saturated Ag/AgCl reference electrode (RE), and a platinum wire counter electrode (CE). The BiOI electrodes were washed thoroughly with Milli-Q water and dried at ambient condition. To convert BiOI to $BiVO_4$, ~200 μL of DMSO solution containing 0.2 M $VO(acac)_2$ was dropped onto the BiOI electrode (2 cm × 1 cm) surface and then annealed at 450°C for 2 h. Finally, residual V_2O_5 on the $BiVO_4$ surface was removed by soaking them in 1 M NaOH solution for 30 min. The resulting bare $BiVO_4$ electrodes were washed thoroughly with Milli-Q water and dried at room temperature. The borate treatment was performed by a simple immersion of $BiVO_4$ in a 0.5 M borate buffer solution at pH 9.3 for 12 h. The resulting B-$BiVO_4$ was washed with Milli-Q water and dried at room temperature.

Photoelectrochemical Measurements

All photoelectrochemical measurements were performed at room temperature using a CHI 650E electrochemical workstation in a three-electrode photoelectrochemical cell. The $BiVO_4$ photoanodes, a saturated Ag/AgCl (3.5 M KCl, 0.197 V vs. NHE), and a Pt wire mesh were configured as WE, RE, and CE, respectively. The recorded potential versus Ag/AgCl ($E_{Ag/AgCl}$) was converted against reversible hydrogen electrode (RHE) according to the Nernst equation ($E_{RHE} = E_{Ag/AgCl} + 0.197 + 0.059 \times pH$). PEC measurements under illumination were conducted with a NEWPORT LCS-100 solar simulator (type 94011A-ES, a 100 W Xenon arc lamp with an AM 1.5G filter) to gain simulated AM 1.5G solar illumination. All $BiVO_4$ photoanodes were illuminated from the FTO (back) side and illuminated areas were 0.25 cm^{-2}. The 0.5 M sodium borate buffer solution (pH=9.3) with or without 0.5 M sodium sulfite (Na_2SO_3) as a hole scavenger was used as the electrolyte. The linear scan voltammetry (LSV) with the scan rate of 10 $mV \cdot s^{-1}$ was used for the dark and photocurrent measurements. The ABPEs of $BiVO_4$ photoanodes were calculated from *J-V* curves according to the equation: $ABPE\ (\%)=(J_{light}-J_{dark})\times(1.23-V_{RHE})/P_{light}\times 100\%$. The IPCE measurement was performed under monochromatic light by a light source ASB-XE-175 (Spectral Products) illumination through a monochromator (Spectral Products CM110). For electrochemical impedance spectroscopy (EIS) measurement, a sinusoidal voltage pulse of 10 mV amplitude was applied on a bias voltage of 0.7 V_{RHE} with frequencies ranging from 100 kHz to 0.01 Hz. For KIE experiments, a pH meter (FiveEasy F20, METTLER TOLEDO) was used for all pH measurements. The pD values were adjusted using NaOD or DCl in accordance with the relation pD = pH_{read} + 0.4. At least three replicates were performed for KIE measurements at room temperature. Mott-Schottky (MS) spectra were measured in a 0.5 M borate buffer (pH 9.3) in the dark (potential window: 0.1~0.4 V_{RHE}, increment: 10 mV, and frequency: 1 kHz). To determine Faradaic efficiency (FE), gastight cell was used, and the solution was degassed by bubbling Helium for 0.5 h. The electrolysis was carried out with a constant potential at 0.7 V_{RHE}, and the actual amount of O_2 evolution was determined by gas chromatography (GC-2014 SHIMADZU) every c.a. 15 min. The theoretical amount of produced O_2 can be obtained by converting the charge passed to μmol gas according to Faraday's Law. The Faradaic efficiency was calculated according to the equation: FE (%) = O_2(actual)/O_2(theoretical) × 100%.

Physical Characterization

X-ray diffraction (XRD) measurements were carried out on Bruker D5000 X-ray diffraction diffractometer with Cu Kα radiation (λ = 1.5406 Å). The X-ray photoelectron spectroscopy (XPS) measurements were performed on a PHI 5500 spectrometer using a monochromatized Al Kα radiation (1487 eV). Scanning Electron Microscope (SEM, JEOL JSM 7401) and Transmission Electron Microscopy (TEM, JEOL JEM2100F) were also used for the morphology and structural characterization of $BiVO_4$ photoanodes. Raman measurements were carried out on a Witec Alpha300 RAS Raman microscope with green laser (532 nm) for excitation. Near edge X-ray absorption fine structure (NEXAFS) spectroscopy measurements were performed at the Elettra synchrotron radiation facility in Trieste, Italy.

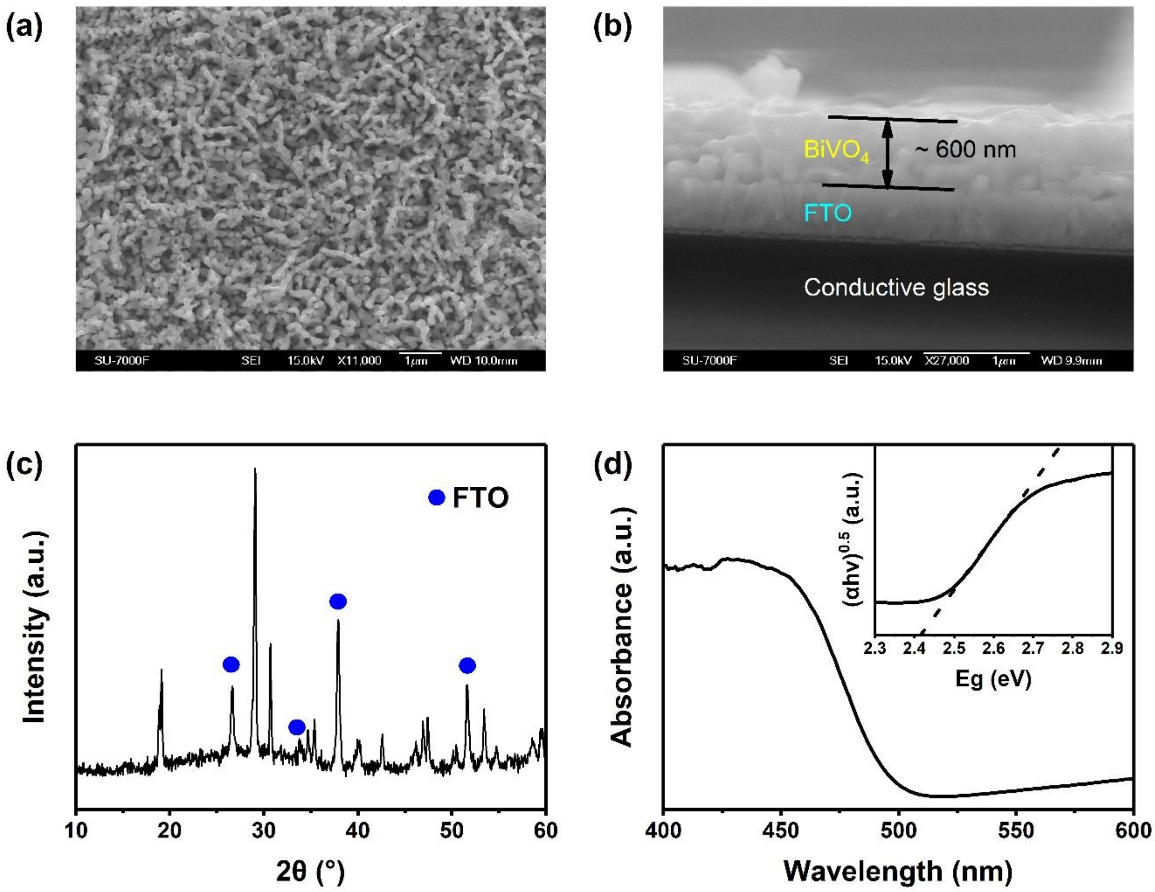

Figure S1. The top (a) and cross-sectional (b) SEM images, XRD spectrum (c) and UV-Vis spectrum (d) of the bare $BiVO_4$ photoanode.

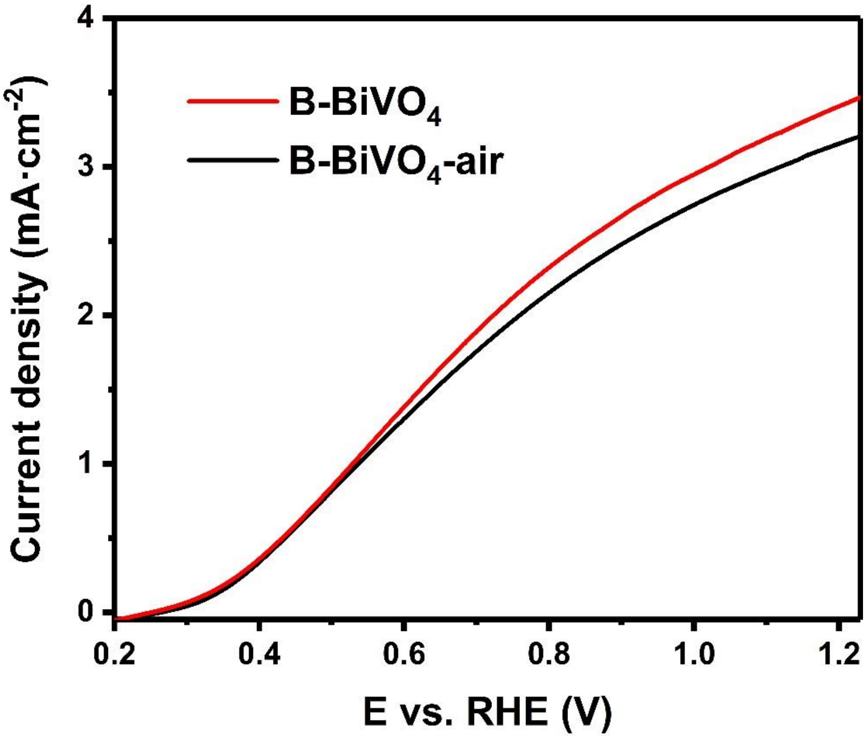

Figure S2. *J* − *V* curves of B-$BiVO_4$ tested before and after free-standing for 24 h at room temperature.

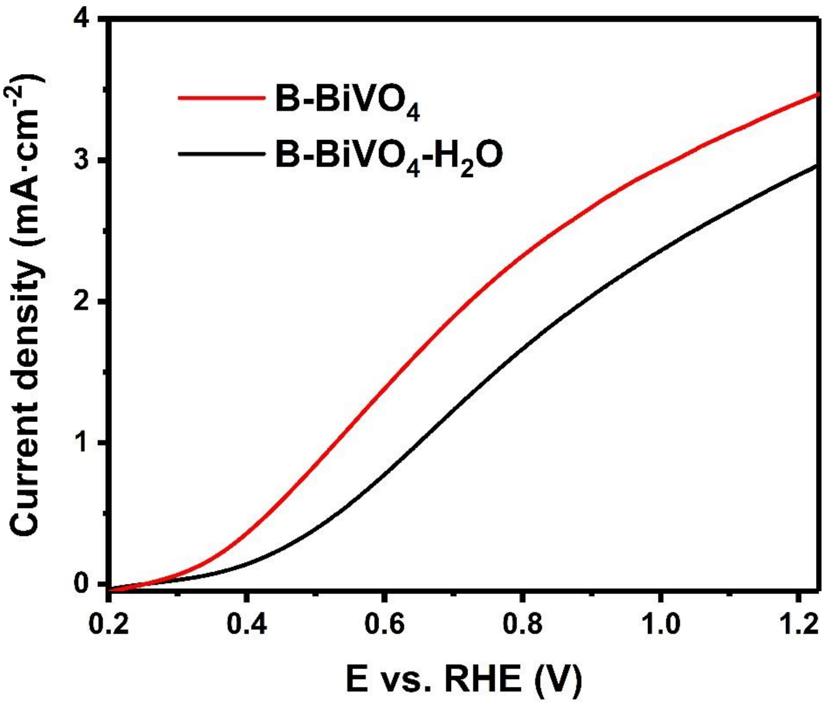

Figure S3. *J* − *V* curves of B-$BiVO_4$ photoanodes after immersion in Milli Q water for overnight.

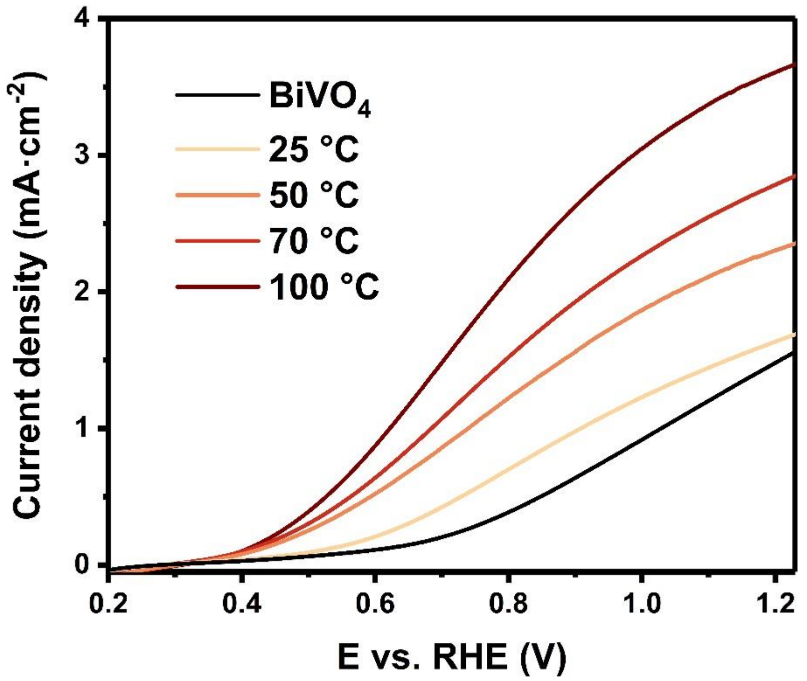

Figure S4. *J* − *V* curves of B-$BiVO_4$ photoanodes treated in a 0.5 M borate buffer (pH 9.3) at different temperatures for 25 min.

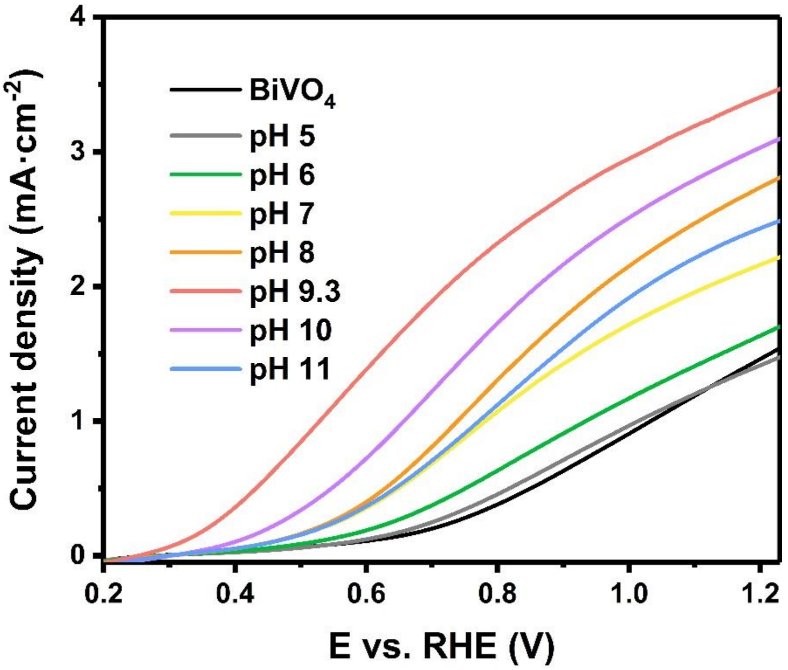

Figure S5. *J* − *V* curves of B-$BiVO_4$ photoanodes treated in a 0.5 M borate buffer at different pH values.

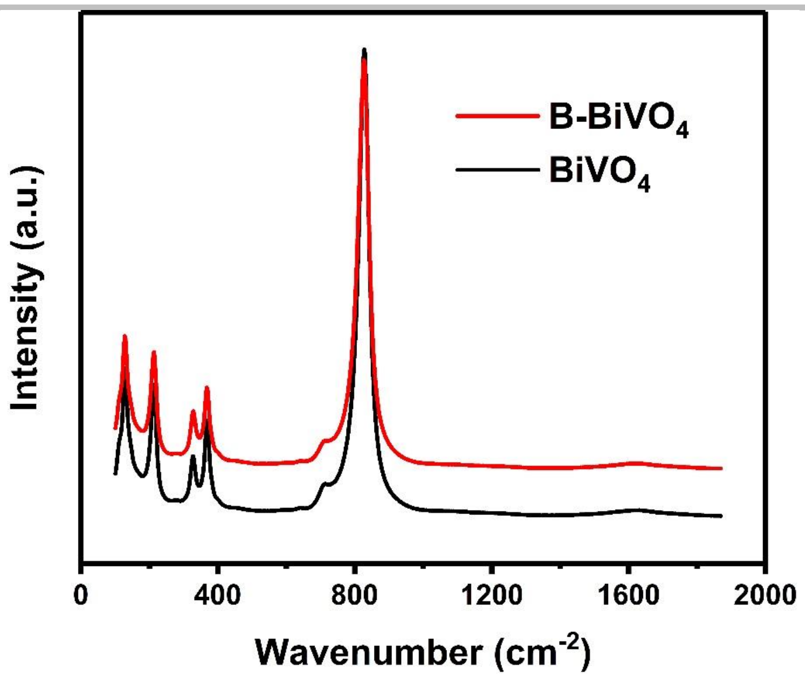

Figure S6. Raman spectra of $BiVO_4$ and B-$BiVO_4$.

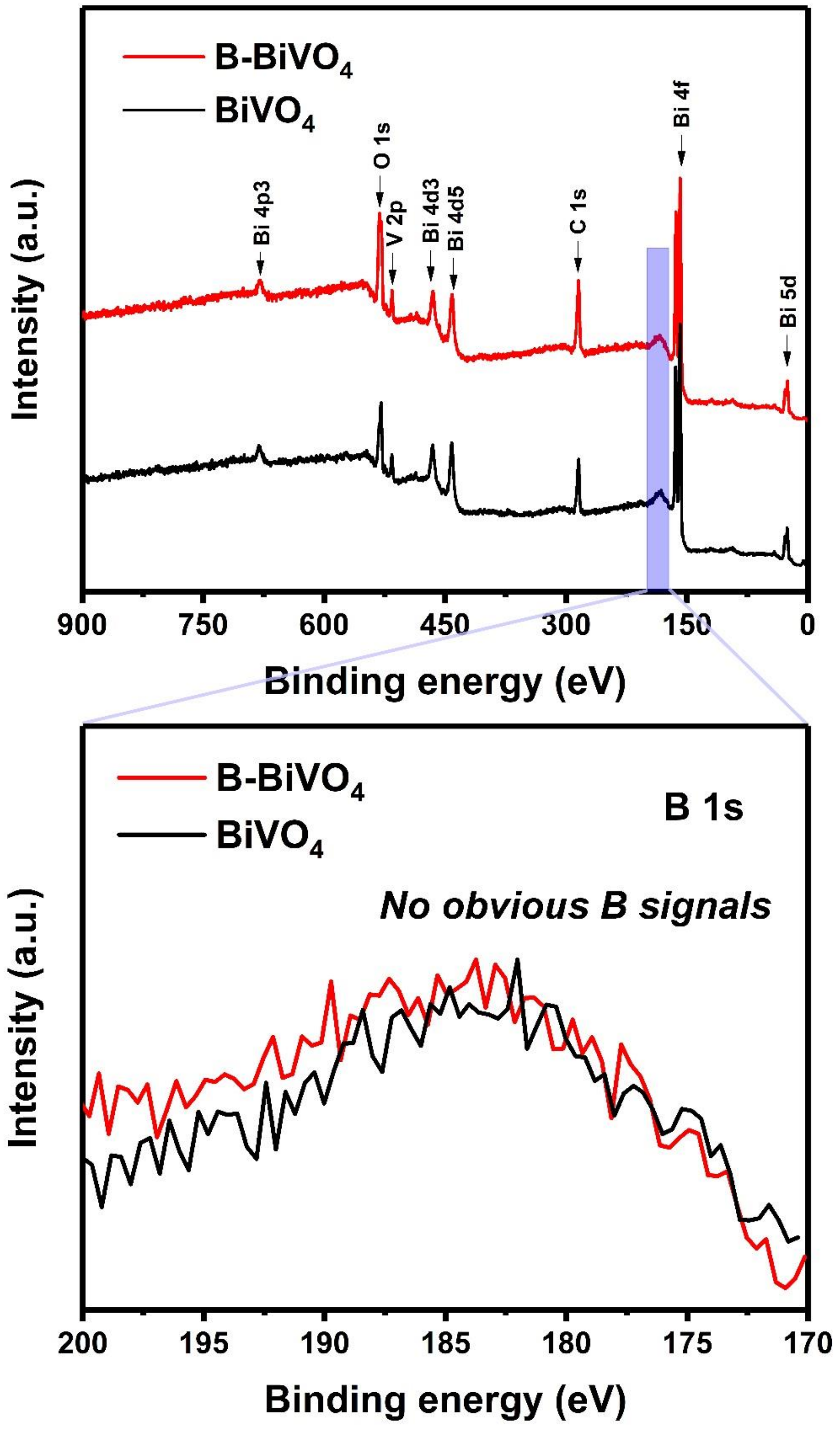

Figure S7. XPS survey spectra and high resolution XPS spectra at the B 1s edges for bare $BiVO_4$ and B-$BiVO_4$ photoanodes.

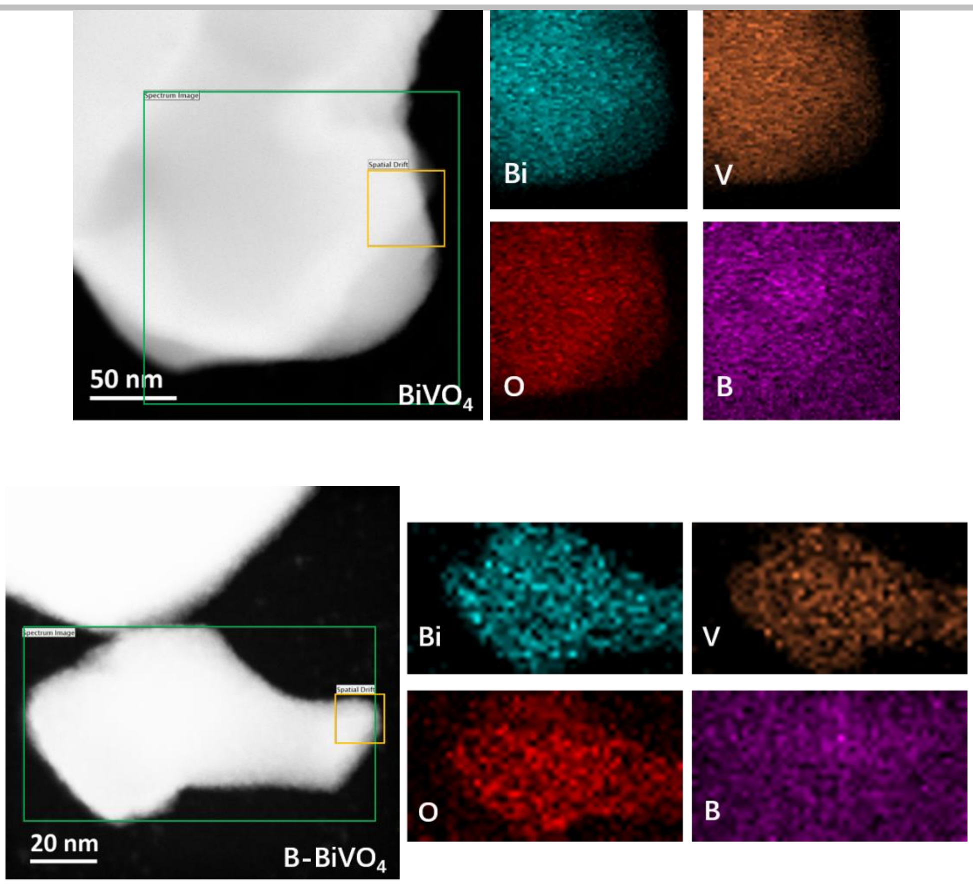

Figure S8. Dark field TEM images and the corresponding elemental mappings of bare $BiVO_4$ and B-$BiVO_4$ photoanodes: Bi (cyan), V (orange), O (red), and B (purple).

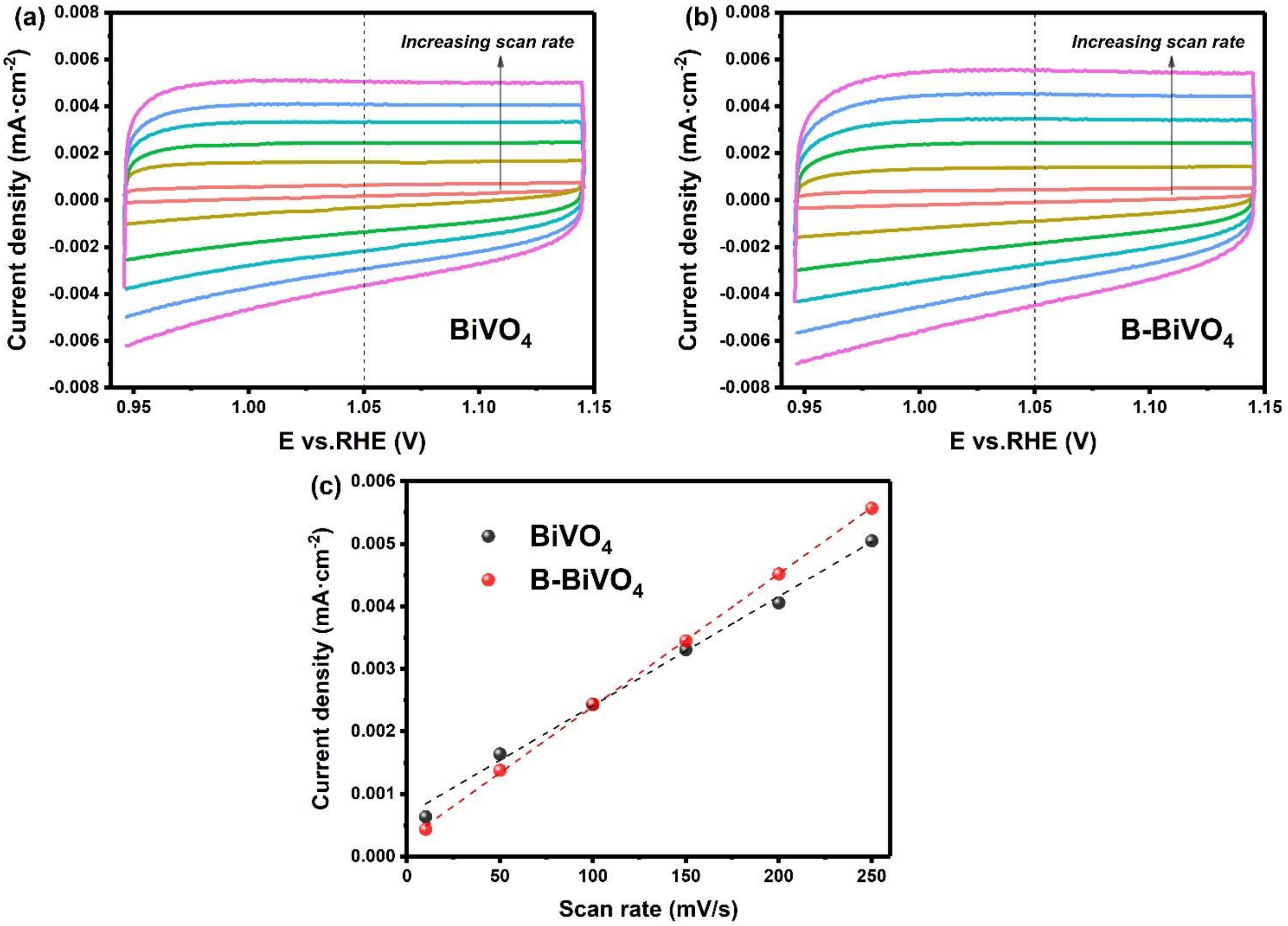

Figure S9. Cyclic voltammograms (recorded in the dark) for (a) $BiVO_4$ and (b) B-$BiVO_4$ at different scan rates (10, 50, 100, 150, 200, and 250 mV/s) in a potential range without Faradaic processes. c) Charging current densities recorded at 1.05 V_{RHE} at different scan rates: the slope reflects the capacitance, which is proportional to the electrochemically active surface area.

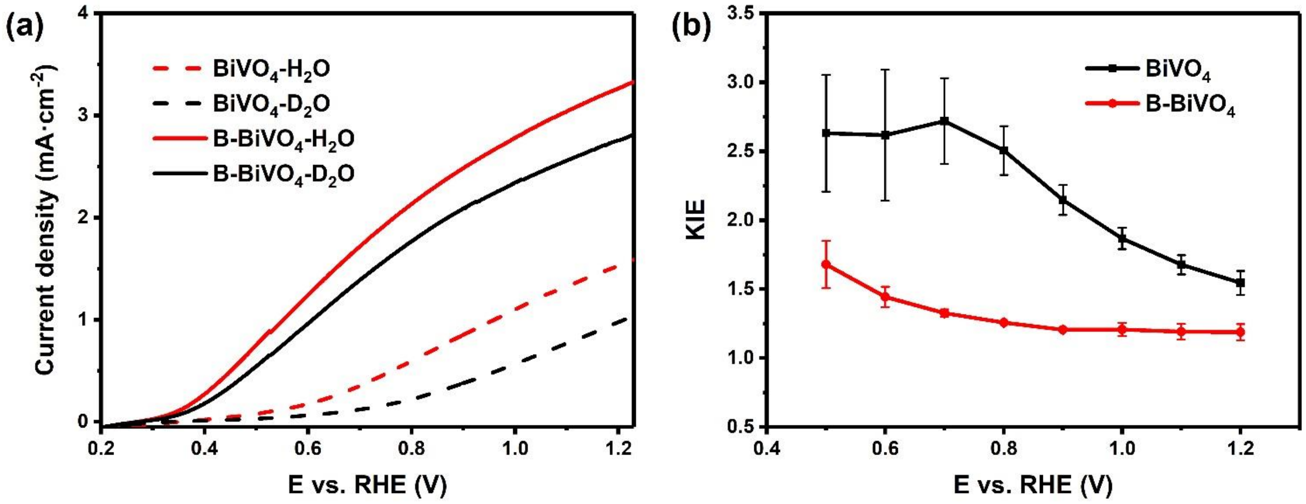

Figure S10. a) *J* – *V* curves of $BiVO_4$ and B-$BiVO_4$ photoanodes in 0.5 M borate H_2O and D_2O solutions, respectively. The pD values were obtained according to the relation pD = pH_{read} + 0.4. b) the kinetic isotope effect (KIE) values of $BiVO_4$ and B-$BiVO_4$ calculated from the photocurrent density ratio in H_2O and D_2O solutions ($KIE = J_{H2O}/J_{D2O}$).

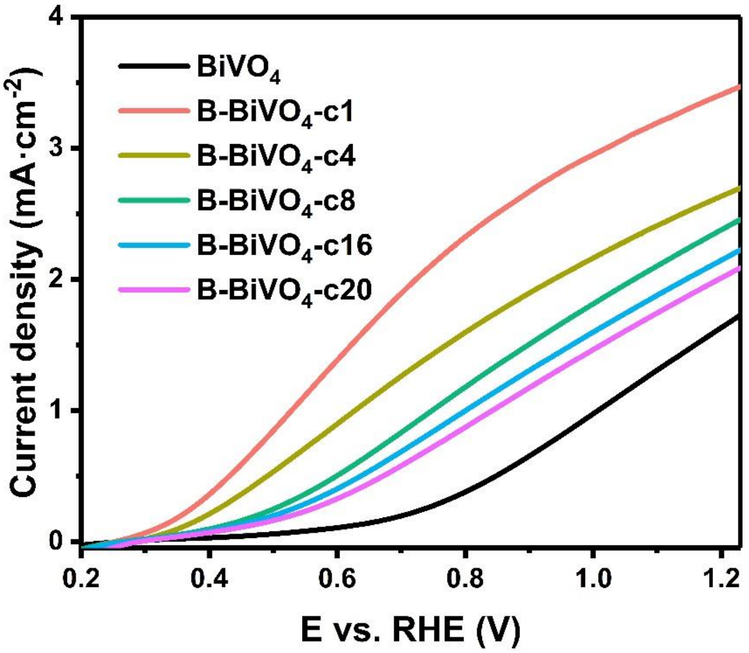

Figure S11. Stability of the B-$BiVO_4$ under photoelectrochemical performance tested by 20 cycles of LSV scanning.

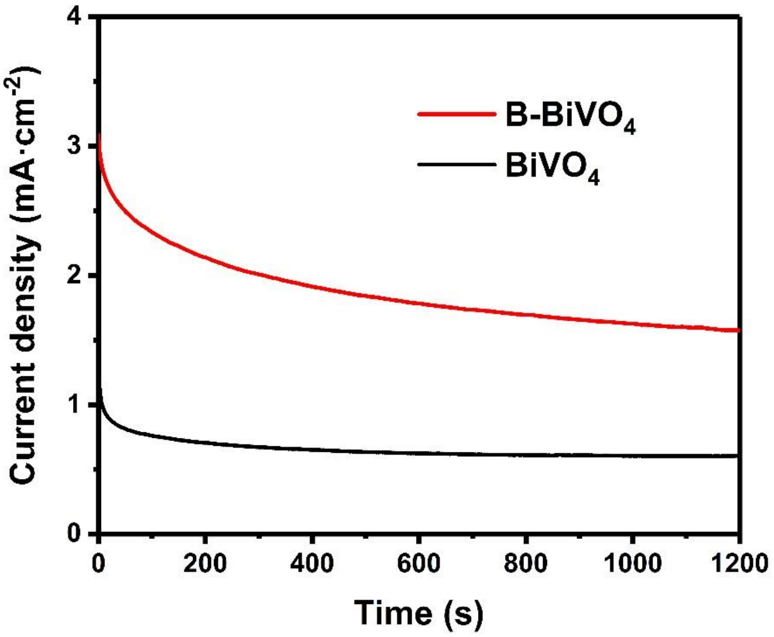

Figure S12. *J-t* curves of $BiVO_4$ and B-$BiVO_4$ photoanodes measured at 1.0 V_{RHE}.

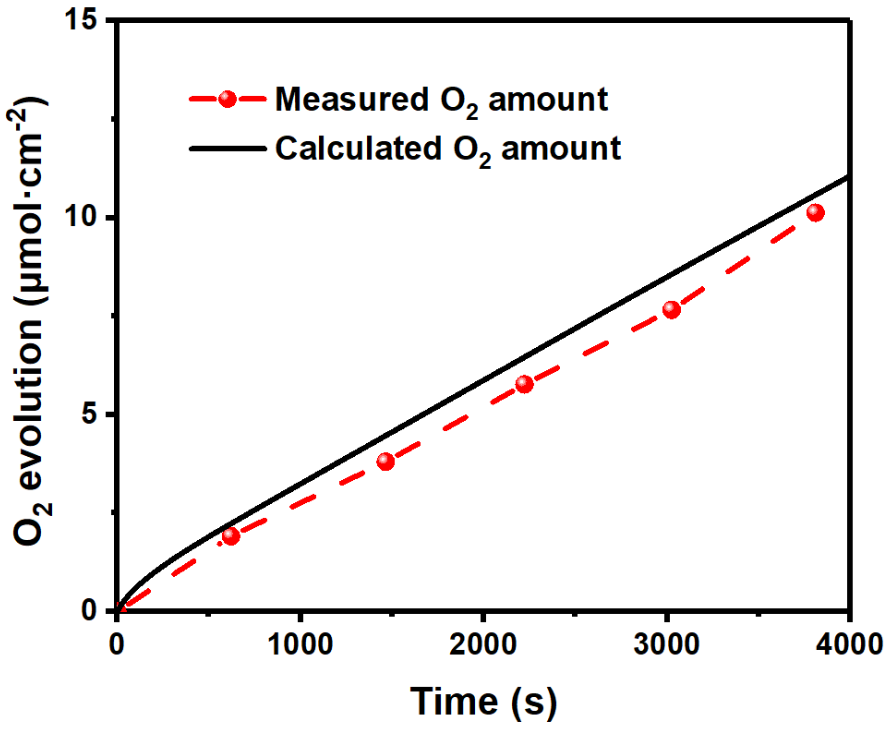

Figure S13. Oxygen evolutions detected by gas chromatography and calculated from photocurrent during the photolysis of B-$BiVO_4$ photoanode at 0.7 V_{RHE}.

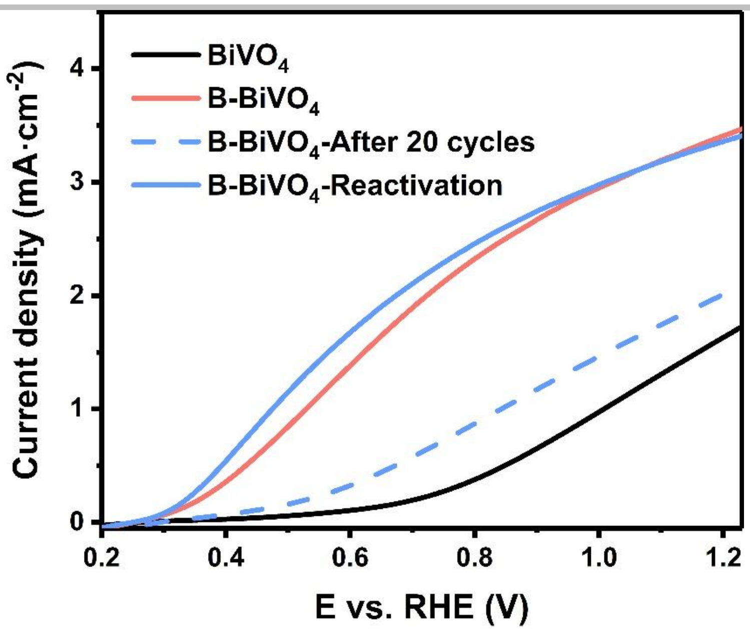

Figure S14. *J − V* curves of $BiVO_4$ photoanodes: bare $BiVO_4$, B-$BiVO_4$ at the 1st cycles of LSV scanning; B-$BiVO_4$ after 20 cycles of LSV scanning; and B-$BiVO_4$ after recovery by further immersing in a 0.5 M borate buffer (pH 9.3) for 12h. Herein, all *J-V* curves are from PEC measurements on the same $BiVO_4$ substrate.

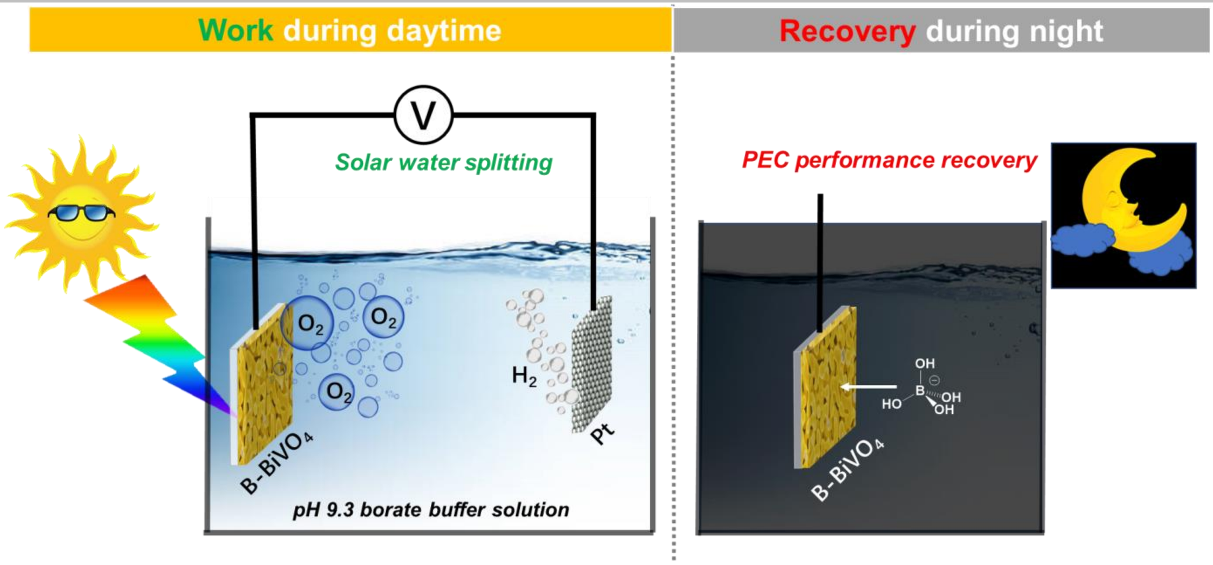

Figure S15. Schematic diagram shows the working pattern of a proposed self-healing PEC cell.

Table S1. PEC performance of $BiVO_4$ photoanodes.

$BiVO_4$ photoanode	J_{ph} @1.23 V_{RHE} ($mA \cdot cm^{-2}$)	Onset potential (V_{RHE})	Test condition[a]	Year
**B-$BiVO_4$**	**3.5**	**0.32**	**0.5 M NaBi, pH 9.3**	**This work**
Co-Pi/1% W:$BiVO_4$ [2]	3.6	0.25	0.1 M KPi, pH 7.3	2013
NiOOH /FeOOH/$BiVO_4$ [1]	4.45	0.23	0.5 M KPi, pH 7	2014
WO_3/W:$BiVO_4$ nanowires [3]	3.1	*0.55	0.5 M KPi, pH 8	2014
Co_3O_4/$BiVO_4$ [4]	2.71	*0.5	0.5 M KPi, pH 7	2015
SnO_2/$BiVO_4$ [5]	2.62	0.67	0.5 M KPi, pH 7	2017
Co-Pi/ZnO/$BiVO_4$ [6]	3.47	0.3	0.3 M Na_2SO_4, pH 7.5	2017
NiB/$BiVO_4$ [7]	3.5	0.25	0.5 M KBi, pH 9.2	2017
β-FeOOH/$BiVO_4$ [8]	4.3	*0.4	0.2M Na_2SO_4, pH 7	2018
Au-Mo:$BiVO_4$ [9]	2.8	0.63	0.5 M KPi, pH 7.4	2018
CoOOH/$BiVO_4$ [10]	4.0	0.25	0.2 M KPi+0.5M Na_2SO_4, pH 7	2018
Co (salophen-H)/$BiVO_4$ [11]	3.89	*0.2	0.1 M KPi, pH 7	2018
$CoPO_3$/pGO/LDH/$BiVO_4$ [12]	4.45	0.17	1.0 M KBi, pH 9	2018
poly-1/Vpa/Al_2O_3/$BiVO_4$ [13]	*2.9	0.3	0.1 M Na_2SO_4, pH 7	2019
Ni:FeOOH/Zn:$BiVO_4$/Mo:$BiVO_4$ [14]	2.7	*0.26	0.5 M KPi, pH 7	2019
Co–Pi/PDA/$BiVO_4$ [15]	2.47	*0.6	0.5 M Na_2SO_4, pH 7	2019
NiOOH/BP/$BiVO_4$ [16]	4.48	*0.25	0.5 M KPi, pH 7.1	2019

[a] NaBi= sodium borate; KPi= potassium phosphate; KBi= potassium borate.

* Photocurrent density or onset potential was estimated from the *J-V* curves in the corresponding reference paper.

Table S2. PEC performance of $BiVO_4$ photoanodes without doping or loading catalysts.

$BiVO_4$ photoanode	J_{ph} @1.23 V_{RHE} ($mA \cdot cm^{-2}$)	Onset potential (V_{RHE})	Test condition[a]	Year
**B-$BiVO_4$**	**3.5**	**0.32**	**0.5M NaBi, pH 9.3**	**This work**
H_2 treated $BiVO_4$ [17]	*2.4	*0.5	0.5 M Na_2SO_4, pH 6.8	2013
N_2 treated $BiVO_4$ [18]	*2.8	*0.4	0.5 M KPi, pH 7.2	2015
Photocharged $BiVO_4$ [19]	3.3	*0.4	0.1M KPi, pH 7.2	2016
UV-treated $BiVO_4$ [20]	1.2	0.42	0.1 M KPi, pH 7	2016
Electrochemically treated $BiVO_4$ [21]	2.5	*0.35	1 M KBi pH 9.5	2017
Photocharged $BiVO_4$ [22]	4.3	0.25	0.1 M PBA, pH 10	2017
[001]-oriented $BiVO_4$ (p-BVO) [23]	3.9	0.6	0.5 M KPi, pH 7	2018
Disordered layer $BiVO_4$ [24]	2.3	0.65	0.5 M KPi, pH 7	2018

[a] NaBi= sodium borate; KPi= potassium phosphate; PBA= phosphate-borate-acetate; KBi= potassium borate.

* Photocurrent density or onset potential was estimated from the J-V curves in the corresponding reference paper.

References

[1] T. W. Kim, K.-S. Choi, *Science* **2014**, *343*, 990-994.
[2] F. F. Abdi, L. Han, A. H. Smets, M. Zeman, B. Dam, R. Van De Krol, *Nat. Commun.* **2013**, *4*, 2195.
[3] P. M. Rao, L. Cai, C. Liu, I. S. Cho, C. H. Lee, J. M. Weisse, P. Yang, X. Zheng, *Nano Lett.* **2014**, *14*, 1099-1105.
[4] X. Chang, T. Wang, P. Zhang, J. Zhang, A. Li, J. Gong, *J. Am. Chem. Soc.* **2015**, *137*, 8356-8359.
[5] S. Byun, G. Jung, S.-Y. Moon, B. Kim, J. Y. Park, S. Jeon, S.-W. Nam, B. Shin, *Nano Energy* **2018**, *43*, 244-252.
[6] J.-S. Yang, J.-J. Wu, *Nano Energy* **2017**, *32*, 232-240.
[7] K. Dang, X. Chang, T. Wang, J. Gong, *Nanoscale* **2017**, *9*, 16133-16137.
[8] B. Zhang, L. Wang, Y. Zhang, Y. Ding, Y. Bi, *Angew. Chem. Int. Ed.* **2018**, *57*, 2248-2252; *Angew. Chem.* **2018**, *130*, 2270–2274.
[9] J. K. Kim, X. Shi, M. J. Jeong, J. Park, H. S. Han, S. H. Kim, Y. Guo, T. F. Heinz, S. Fan, C.-L. Lee, J. H. Park, X. Zheng, *Adv. Energy Mater.* **2018**, *8*, 1701765.
[10] F. Tang, W. Cheng, H. Su, X. Zhao, Q. Liu, *ACS Appl. Mater. Interfaces* **2018**, *10*, 6228-6234.
[11] Y. Liu, Y. Jiang, F. Li, F. Yu, W. Jiang, L. Xia, *J. Mater. Chem. A* **2018**, *6*, 10761-10768.
[12] S. Ye, C. Ding, R. Chen, F. Fan, P. Fu, H. Yin, X. Wang, Z. Wang, P. Du, C. Li, *J. Am. Chem. Soc.* **2018**, *140*, 3250-3256.
[13] W. Jiang, X. Yang, F. Li, Q. Zhang, S. Li, H. Tong, Y. Jiang, L. Xia, *Chem. Commun.* **2019**, *55*, 1414-1417.
[14] J. M. Lee, J. H. Baek, T. M. Gill, X. Shi, S. Lee, I. S. Cho, H. S. Jung, X. Zheng, *J. Mater. Chem. A* **2019**, *7*, 9019-9024.
[15] Y. Gao, W. Fan, K. Qu, F. Wang, P. Guan, D. Xu, H. Bai, W. Shi, *New J. Chem.* **2019**.
[16] K. Zhang, B. Jin, C. Park, Y. Cho, X. Song, X. Shi, S. Zhang, W. Kim, H. Zeng, J. H. Park, *Nat. Commun.* **2019**, *10*, 2001.
[17] G. Wang, Y. Ling, X. Lu, F. Qian, Y. Tong, J. Z. Zhang, V. Lordi, C. Rocha Leao, Y. Li, *J. Phys. Chem. C* **2013**, *117*, 10957-10964.
[18] T. W. Kim, Y. Ping, G. A. Galli, K.-S. Choi, *Nat. Commun.* **2015**, *6*, 8769.
[19] B. J. Trześniewski, W. A. Smith, *J. Mater. Chem. A* **2016**, *4*, 2919-2926.
[20] L. Tengfei, H. Jingfu, P. Bruno, B. C. P., *Angew. Chem. Int. Ed.* **2016**, *55*, 1769-1772; *Angew. Chem.* **2016**, *128*, 1801-1804.
[21] S. Wang, P. Chen, J.H. Yun, Y. Hu, L. Wang, *Angew. Chem. Int. Ed.* **2017**, *56*, 8500-8504; *Angew. Chem.* **2017**, *129*, 8620-8624.
[22] B. J. Trześniewski, I. A. Digdaya, T. Nagaki, S. Ravishankar, I. Herraiz-Cardona, D. A. Vermaas, A. Longo, S. Gimenez, W. A. Smith, *Energy Environ. Sci.* **2017**, *10*, 1517-1529.
[23] H. S. Han, S. Shin, D. H. Kim, I. J. Park, J. S. Kim, P.-S. Huang, J.-K. Lee, I. S. Cho, X. Zheng, *Energy Environ. Sci.* **2018**, *11*, 1299-1306.
[24] J. K. Kim, Y. Cho, M. J. Jeong, B. Levy-Wendt, D. Shin, Y. Yi, D. H. Wang, X. Zheng, J. H. Park, *ChemSusChem* **2018**, *11*, 933-940.

Author Contributions

Q. Meng and B. Zhang designed the experiments, fabricated the PEC devices, performed the photoelectrochemical measurements and wrote the paper. B. Zhang and L. Sun supervised the project. L. Fan carried out the XRD, SEM and TEM characterization. H. Liu and K. Edström executed and analyzed the XPS measurements. M. Cuartero, R. de Marco and G.A. Crespo executed, analyzed and discussed the synchrotron-based measurements. M. Valvo carried out the Raman characterization. All authors contributed to the scientific discussion and revision of the paper.
